# Mitigating urinary incontinence condition using machine learning

**DOI:** 10.1186/s12911-022-01987-3

**Published:** 2022-09-17

**Authors:** Haneen Ali, Abdulaziz Ahmed, Carlos Olivos, Khaled Khamis, Jia Liu

**Affiliations:** 1 Health Services Administration Program & Department of Industrial and Systems Engineering, Auburn University, 351 W Thach Concourse, 7080 Haley Center, Auburn, AL 36849 USA; 2 Department of Health Services Administration, School of Health Professions, The University of Alabama at Birmingham, 1720 University Blvd, Birmingham, AL 35294 USA; 3 Department of Industrial and Systems Engineering, Samuel Ginn College of Engineering, Auburn University, 345 W Magnolia Ave, Auburn, AL 36849 USA; 4 Departamento de Ingeniería Industrial, Universidad Católica del Norte, Antofagasta, Chile

**Keywords:** Machine learning, Urinary incontinence, Urination, Bladder voiding

## Abstract

**Background:**

Urinary incontinence (UI) is the inability to completely control the process of releasing urine. UI presents a social, medical, and mental issue with financial consequences.

**Objective:**

This paper proposes a framework based on machine learning for predicting urination time, which can benefit people with various degrees of UI.

**Method:**

A total of 850 data points were self-recorded by 51 participants to investigate how different factors impact urination time. The participants were instructed to record input data (such as the time of consumption and the number of drinks) and output data (i.e., the time the individual urinated). Other factors, such as age and BMI, were also considered. The study was conducted in two phases: (1) data was prepared for modeling, including missing values, data encoding, and scaling; and (2) a classification model was designed with four output classes of the next urination time: <  = 30 min, 31–60 min, 61–90 min, > 90 min. The model was built in two steps: (1) feature selection and (2) model training and testing. Feature selection methods such as lasso regression, decision tree, random forest, and chi-square were used to select the best features, which were then used to train an extreme gradient boosting (XGB) algorithm model to predict the class of the next urination time.

**Result:**

The feature selection steps resulted in nine features considered the most important features affecting UI. The accuracy, precision, recall, and F1 score of the XGB predictive model are 0.70, 0.73, 0.70, and 0.71, respectively.

**Conclusion:**

This research is the first step in developing a machine learning model to predict when a person will need to urinate. A precise predictive instrument can enable healthcare providers and caregivers to assist people with various forms of UI in reliable, prompted voiding. The insights from this predictive model can allow future apps to go beyond current UI-related apps by predicting the time of urination using the most relevant factors that impact voiding frequency.

## Introduction

Urinary incontinence (UI) is the inability to control the process of releasing urine. There are five forms of UI: (1) stress type, which describes the leaking of urine during intensive pelvic activities, such as coughing or sneezing; (2) urge type, which involves sudden urges followed by involuntary urination; (3) functional type, related to physical or mental impairments that mean using the toilet requires assistance; (4) overflow type, or the inability to completely void one’s bladder; and (5) mixed type, which is a combination of types that makes holding one’s bladder harder or impossible to control [1, 2]. Studies have shown that about 25% of the U.S. population suffers from urinary incontinence [1, 2]. More than 25 million people in the United States experience bladder leakage every day, and around 33 million have overactive bladders, which increases the risk of UI [1, 3]. The actual number could be larger because adults may not report the issue or seek treatment [1, 3].

UI is a socially embarrassing and psychologically distressing disorder [4]. The fear of wetting oneself in public can influence the ability of a UI patient to interact in social and professional situations. As a result, UI can interfere with a patient’s work, schooling, social life, and family stability [4]. UI also presents immediate safety and medical concerns. The lack of certainty about when the next urination will occur can increase the risk of injuries in rushing to the bathroom [5]. Additionally, aging people have a higher likelihood of experiencing adverse events like falling while going to the restroom; they also have a higher chance of developing harmful urinary conditions, such as urinary tract infections or voiding dysfunction [4]. Due to the mental, social, and medical impacts of uncontrolled urination, there is a need to develop a tool that can help predict the time an individual is expected to urinate [6–8].

Predictive models are crucial in optimizing the process of prompted voiding for individuals and caregivers of individuals with UI [6–8]. Prompted voiding involves a person with a compromising urinary or bladder condition being assisted to the bathroom in anticipation of a full bladder [4]. Without a predictive tool, it is impractical and nearly impossible to provide reliable prompted voiding assistance for someone with a form of UI [6–8]. Machine learning (ML) techniques can improve urination prediction for people with UI by bypassing physics-based modeling and formulating complex relationships related to intake and output frequency in a data-driven manner [6–8]. ML can also allow future apps to go beyond current UI-related apps by predicting when someone will need to urinate and determining which factors are essential to predict the next urination. However, proper feature selection and ML functions must be determined to predict urination.

This paper proposes a comprehensive framework for developing a ML model to predict the next urination times for individuals with UI. A total of 51 individuals with UI conditions were recruited and monitored for five days. Different data points were collected from the subjects, such as the amount of liquid intake, and the types of drinks consumed. The time a subject needed to urinate after doing certain activities or drinking was also recorded. Next, the data were modeled to build a predictive model, which was conducted in two phases: data preprocessing and data modeling. Different data preprocessing steps were conducted, including feature encoding, scaling, and missing data treatment. The output feature, which is the time the individual urinated, was converted into three categories: short, medium, and long. The modeling phase involved both training and testing an XGB model. The model was evaluated using five performance measures: accuracy, recall, precision, and F1 score. The contributions of this study are as follows:

- This is the first study that proposes a model to mitigate UI based on predictive analytics.
- This study determines the most important factors associated with urination prediction.
- An interpretation of the factors that affect UI is provided.
- The proposed model is practical and can be turned into a smartphone application that can help individuals with UI conditions.

## Literature review

Different studies have been conducted to investigate the factors associated with UI and how they can be used to assist individuals with UI. Asklund et al. [9] conducted a study to evaluate a treatment of stress type UI (SUI). They conducted a randomized controlled trial comprising over 100 Swedish female subjects with SUI. The subjects used a mobile app that included pelvic floor muscle training (PFMT) exercises to treat SUI. Half (50%) of the women used the app, while the rest did not. The results showed that the women who had access to the PFMT app had lower symptom severity than the women without the app. Nyström et al. [10] provided a follow-up study that used the data published by Asklund et al. [9] to determine the factors associated with successful treatment (i.e., lower SUI symptom severity). Nyström et al. [10] suggested that monitoring the weight control of an individual and PFMT exercises helped to mitigate the effect of SUI. However, their study did not provide a comprehensive approach when evaluating which foods affected urination the most [9, 10].

Nowadays, systems store large datasets that provide insightful information when using data analytics methods. ML can detect hidden insights and trends in large amounts of data helping to predict and classify certain events. This capability is helpful in healthcare when diagnosing heterogeneous data, especially for less experienced physicians [11]. Thus, these techniques can support physician to improve their accuracy and efficiency in prognosis, diagnosis, treatment, and clinical workflows [12]. For example, these techniques have been used to diagnose heart and diabetic diseases, breast cancer, and thyroid disorders, offering accuracies above 75% [13]. In the context of this research, there are no conventional methods to predict the time a person needs to urinate. Moreover, finding insights without data analytics techniques could result in unsuccessful results due to data structure. Thus, ML can provide this information to help address undesirable events for people with UI.

Multiple mobile applications have been developed to mitigate UI problems. The Sit or Squat application was developed to help users find the nearest public restroom anywhere in the United States [14, 15]. BladderPal and UroBladderDiary mobile applications help track urination and consumption [14–16]. Kegel Trainer PFM and Tät help to encourage and instruct users to conduct PFMT [9, 10, 17], and Tät provides additional resources aside from PFMT instructions, such as remedies to help address UI symptoms [18]. While these apps aid in addressing UI, none directly address the uncertainty associated with the likelihood that an individual, particularly one with a form of UI, will have access to a restroom when they need one after drinking or performing physical activity. The literature on current UI and urination apps suggests an increasing need for time management technology to help people with UI anticipate when they will need to urinate [9, 10, 17–21]. Because of the individual factors related to consumption and the presence of comorbidities that influence urination, predictive modeling is necessary to determine the likelihood that urination will occur.

Fechner et al. [6] proposed a prediction model to determine bladder filling levels based on user-tracked fluid intake. The authors used a Gaussian curve-fitting algorithm to predict the filling levels of males without bladder conditions between the ages of 20 and 30. Taku et al. [7] used a mathematical model and linear regression analysis to predict bladder volume in an experimental setting. Tantin et al. [22] used linear discriminant analysis to forecast bladder voiding. The authors used a model based on linear discriminant analysis in lab rats. Raw pressure curves and their corresponding power bands were used as inputs for a linear discriminant analysis classifier.

One of the underlying issues among these studies is their lack of examination of real-world environmental factors that influence human factors in urination. Fechner et al. [6] provided an experimental design that restricted the consumption of alcoholic substances. Studies suggest that certain products, such as alcohol, caffeinated beverages, carbonated drinks, and citrus drinks, are bladder irritants and diuretics, which increase the urgency of urination and thereby worsen UI conditions [23]. As alcohol is found to influence bladder control and the time it takes to fill, a prediction model that does not account for alcohol consumption has little use outside of clinical testing. A design that considers the characteristics of liquids consumed is needed to improve the accuracy of future prediction models and better understand the extent to which the type of liquid consumed impacts urination [6, 24].

One of the disadvantages of prediction models in the literature is that the data used for building models are limited. For example, the models proposed by Tantin et al. [22] did not examine human bladder voiding. Fechner et al.’s [6] prediction model is based on data that only included males between 20 and 30 years old. The purposes of these studies were to address and treat the effects that UI and bladder dysfunction have on the ability of an individual to control their urination. However, the data used in previous studies are insufficient to properly determine the most important factors that affect urination for the broader population.

## Research framework

**Fig. 1:**
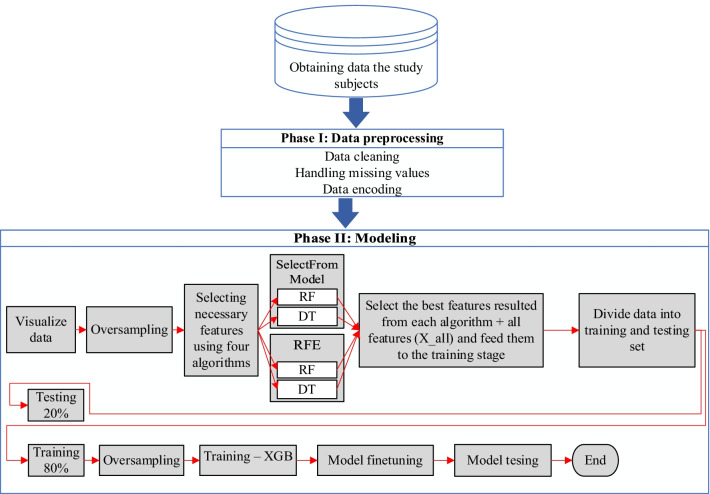
Research framework

The research framework of this study consists of two phases (see Fig. 1). Phase I began after data collection and involved multiple data preprocessing steps applied to prepare the data for modeling. Phase II included the modeling steps, such as feature selection, model training, and testing. Four performance measures were used to evaluate the proposed predictive model: accuracy, precision, recall, and F1 score.

### Data collection and feature generation

This study was approved by the Ethics Committee of Auburn University in accordance with the Declaration of Helsinki (IRB protocol reference: 17-527 EP 1802). Participants were notified about the aims of the project and the data collection process and time. Consent forms were signed by the participants or by their guardians. Participants were notified that no identifiable information would be collected and compensated for their time ($20).

We collected self-recorded information that might impact urination time from 51 participants over five days. The participants were recruited through flyers in medical clinics in Auburn, AL using advertisement boards in town and on-campus and social media platforms. The sample collected reflects a diverse group of people from different demographic groups with diverse diets and lifestyles. Table 1 shows the demographic information of the participants.

**Table 1:** Demographic information of participants by age group

Gender	Age group	No	Age (years)		Weight (kg)		Height (m)		BMI ($\frac{{\bf kg}}{{{\bf m}}^{2}})$	
			Average	SD	Average	SD	Average	SD	Average	SD
Male	Children	2	7.5	3.5	26.0	7.1	1.5	0.2	11.5	0.1
	Youth	13	19.2	1.7	85.2	13.2	1.8	0.2	26.0	3.6
	Adults	12	34.2	10.3	76.1	11.8	1.7	0.1	26.1	2.4
	Seniors	1	71.0	n/a	55.0	n/a	1.6	n/a	21.5	n/a
Female	Children	5	7.6	1.3	25.3	1.6	1.5	0.1	11.9	0.8
	Youth	1	17.0	n/a	99.0	n/a	1.6	n/a	37.3	n/a
	Adults	16	43.5	12.1	77.5	16.4	1.7	0.1	28.2	4.3
	Seniors	1	70.0	n/a	80.0	n/a	1.6	n/a	31.3	n/a
	Total	51	30.7	17.3	72.0	23.2	1.7	0.1	25.0	6.5

**Fig. 2:**
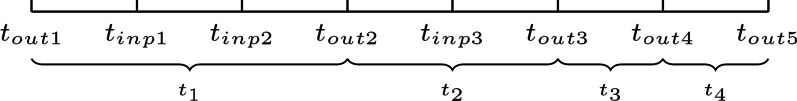
An illustration of an events timeline for a participant in a day

Each day, the participants recorded two types of information related to drinking and urination: input and output. The input information consisted of liquid volume, type of drink, number of drinks, and time of consumption. The output data consisted of the urination time (e.g., when urination occurred; Table 2). Other features were defined and extracted from the self-recorded information. For instance, Fig. 2 shows the event timeline of a participant for one day with three drinks and five urinations. We can define and extract (1) inter-release time ${t}_{i}$ as the time between two urination events; and (2) cumulative volume, which is the sum of all drink volumes during the inter-release time. Demographic data, such as age, gender, weight, height, employment status, exercise level, alcohol consumption, smoking habits, allergies, preexisting conditions, medications, and eating habits, were also collected. The recorded information from the participants generated about 900 data points.

**Table 2:** Potential features from participants’ self-records for the machine learning model

Input data	Type of drinks	Demographic data	Number of drinks	Output data
Liquid volume	Alcohol	Age	Exercise level	Urination time
Number of drinks	Coffee	Youth/adult/senior	Exercise per week	
Time of consumption	Juice	Gender		
	Milk	Weight		
	Soda	Height		
	Water	BMI

The inter-release time is related to the next urination time, so it is the target variable that needs to be predicted to mitigate UI. Since inter-release time is a numerical variable and we have only 900 data points, it is difficult to develop a model that can predict the exact time of urination. Therefore, we converted the output feature (e.g., inter-release time) into four classes: Class 0: <  = 30 min, Class 1: 31–60 min, Class 2: 61–90 min, and Class 3: > 90 min. Then, a classification model was developed to predict the four classes. Whenever the proposed model is run, it can help the subject know when they will need to urinate within one of the four classes.

### Data preprocessing

The initial dataset from 51 participants had around 955 records and 28 features. The dataset included several outliers that were misspelled in the features, such as the number of drinks and liquid volume. We removed the records with values above the 95^th^ percentile of these features. Additionally, data records with unusually large values in the target variable (e.g., inter-release time) were deleted with a threshold of 300 min (5 h). Target variable larger than 600 min (10 h) implied a person did not urinate during the day. Lastly, data records containing a target variable with 0 were removed.

**Fig. 3:**
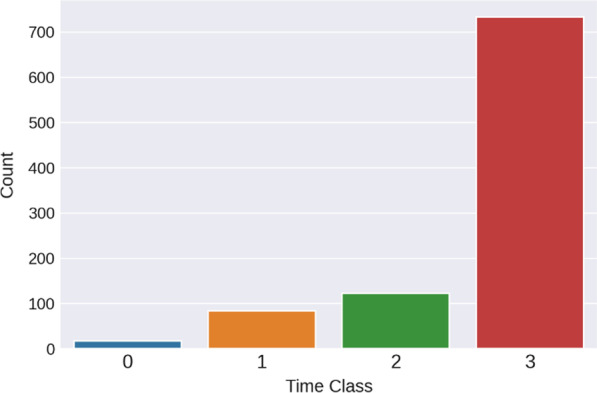
The distribution data in four output classes, showing imbalances in the data

Moreover, since the resulting data had several missing values (see Table 3), three steps were implemented to handle them. In the first step, the feature “Exercise per week” was dropped since it had a high percentage of missing values. Second, k-nearest neighbor (KNN) was utilized as an imputation tool to fill in the missing data for only the numerical features. Data imputation was performed to avoid removing valuable information since the dataset was not large. The KNN imputer is used to determine the number of $k$ neighbors based on Euclidean distances, and the missing values are filled with the average of the *k* neighbors. After filling in the missing values of the numerical features, the rows with missing values among the categorical features were removed. Moreover, data scaling was also conducted. The resulting dataset contains 925 entries and 25 features. Figure 3 shows the output classes after removing the missing data. Notice that the data are significantly imbalanced: Class 4 has the largest number of observations, while Class 1 has the smallest number. In order to mitigate imbalance issues, the training set was oversampled using the Synthetic Minority Oversampling Technique (SMOTE) oversampling method after splitting the dataset into training and testing sets.

**Table 3:** Percentage of missing values

Features	No. of missing values	% of missing values
Exercise per week	150	15.7
Volume	35	3.7
Employment	31	3.2
Vol Inp	9	0.9
Weight	9	0.9
Height	9	0.9
BMI	9	0.9

## Model development

Model development involves two main steps: feature selection and model training and testing. Two main search methods are used to select the most important features: recursive feature elimination (RFE) and select from model (SFM). The RFE and SFM methods are greedy search methods that select features recursively. In the SFM model, a threshold is set, and then any feature below the threshold is removed from the model. In the RFE, a model starts with all features and keeps removing less important features. Both SFM and RFE are conducted with a ML algorithm. In this study, the two methods are implemented using two algorithms: decision tree (DT) and Random Forest (RF). DT is a non-parametric supervised learning method used for classification [25], regression [26], and feature selection [27]. RF is like DT except that many trees are conducted, then a class with major votes is considered as the correct class. The feature importance in both DT and RF are decided based on the Gini index (Eq. 1) [28], where ${p}_{j}$ is the percentage of the samples labeled with class $j$ in the sample set $D$.

1 $$Gini\left(D\right)=1- \sum_{j=1}^{n}{p}_{j}^{2}$$

A data group can result from the DT and SFM search method with the Gini index. This applies to the other data groups that resulted from RF and RFE, and DT and RFE. The features obtained from the feature selection step are used to build a classification model based on XGB, a gradient boosting machine method. XGB is ensemble learning that can be used for classification [29], regression [30], and feature selection [31]. This algorithm builds learners in parallel and trains the models based on residual errors from a previous learner. In XGB, trees are generated iteratively, in which information from a previous tree is used to enhance the quality of the current tree. A higher weight is given to the misclassified instances from previous trees. Hence, XGB trains weak learners. XGB has many parameters, including learning rate, maximum depth, number of estimators, and regularization parameters. To fine-tune XGB parameters, the grid search (GS) approach is used. In GS, different values for XGB’s parameters are set and then XGB is trained and tested based on the combination of all values of the parameters. Four performance measures are used to evaluate the XGB model: accuracy, precision, recall, and F1 score. To avoid overfitting, the data is split into training and testing sets. The training set is used to train the XGB model using cross-validation. The performance of the cross-validation is compared with the performance of the model on the testing set.

## Experimental results

The results of this section were performed using the libraries XGBoost and scikit-learn coded in Python 3.8. Seven data groups result from the feature selection step. Each data group is used to train and test prediction models based on XGB. Also, all features are used to build a model. Table 4 presents the feature selection results. To perform the feature selection, we used SFM and RFE with Lasso regression (Lasso_SFM, Lasso_RFE), DT (DT_SFM, DT_RFE), and RF (RF_SFM, RF_RFE). Chi-square with kbest selection (chi_SKB) was also used. The check mark means that a feature selection step selects a feature. Models that use RFE selected more features as well as chi_SKB. Each data group is split into training and testing groups. K-fold validation is used on the training test to check over-fitting. Regarding accuracy, precision, recall, and F1 score, XGB with “chi_SKB” and “RF_RFE” performed the best with values above 70%. The validation accuracy is within 10% of the testing accuracy (77%), which means that our proposed XHB model did not have overfitting. Thus, these results suggest that the proposed model can be considered an acceptable prediction model for urination time predictions.

**Table 4:** Feature selection results

Feature	Lasso_SFM	DT_SFM	RF_SFM	chi_SKB	DT_RFE	RF_RFE	Lasso_RFE	Total
Water	√			√	√	√	√	5
Volume		√	√	√	√	√		5
VolInp-V of 1st drink		√	√	√	√	√		5
TimeInp-1st output and 2st drink/time		√	√	√	√	√		5
BMI		√	√	√	√	√		5
Age		√	√	√	√	√		5
Alcohol	√				√	√	√	4
Tea	√			√			√	3
Ndrinks				√		√	√	3
Alcoholic	√			√			√	3
Soda	√						√	2
Smoking	√						√	2
Level_exercise					√	√		2
Juice	√						√	2
Gender	√				√			2
Employment					√	√		2
Coffee				√			√	2
Milk							√	1

**Fig. 4:**
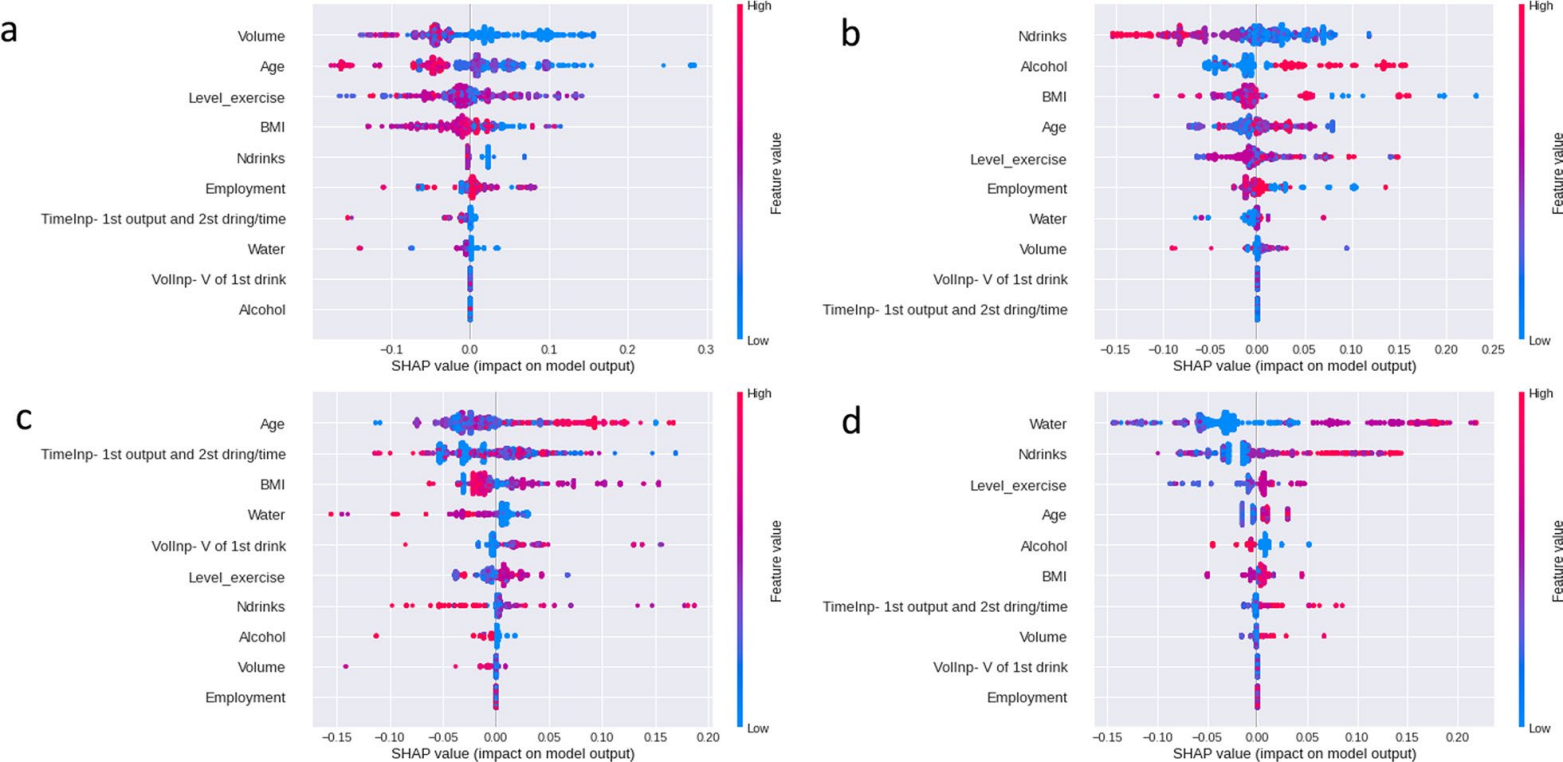
SHAP values for all four classes

We analyzed the effect of the selected features on the model outcome using the Shapley additive explanations (SHAP) value. SHAP is an approach based on game theory to explain the impact of a feature’s value on the response variable being either positive or negative [32]. Figure 4a shows the effects of the model features selected for Class 0. The volume had the highest impact on the next urination time, and alcohol had the lowest effect on this class. The volume feature is inversely proportional to Class 0. Thus, a small volume increases the chances of being classified as Class 0. The volume feature must be analyzed along with age. It is known that a person with low age, i.e., children, can hold urination in less time. Moreover, it is known that they consume less volume. Thus, people of low age are assigned to this class. The above is confirmed with the BMI. A higher BMI decreases the chance of being classified as Class 0.

Figure 4b shows the effects of the model features selected in Class 1. The results for Class 1 suggest that the number of drinks had the highest impact on the response variable, followed by alcohol and BMI. When drinking fewer drinks that include alcohol, the subject is likely to be assigned as Class 1. The above is confirmed with the feature age. Aging increases the chances of being classified as Class 1. It is known that since alcohol acts as a diuretic drink, fewer alcohol drinks decrease the time between urination. Moreover, adults are generally people that drink alcohol.

Figure 4c shows the effects of the model features selected in Class 2. The results for Class 2 suggest that age affects the response variable positively. Aging increases the chances of being classified in this class. As in the previous cases, people of more age can hold longer. Thus, it is very likely to be assigned to this class. Even though the variable water has a negative impact on this class, it is known that water does not act as a diuretic as other drinks such as coffee or alcohol.

Figure 4d shows the effects of the model features selected in Class 3. The results for Class 3 suggest that water had the highest positive impact, with the most significant feature value among the selected characteristics. The number of drinks was the second most important determinant and positively impacted the model output. Water intake and the number of drinks have the greatest impact in increasing the chance of being assigned to this class. Aging is also found to increase the chance of being assigned to this class. People who drink more extend their consumption over a long time and, as a result, urinate more frequently.

### Comparison of methods

We compared the XGB model with all combinations of models and feature selection methods to other ML algorithms such as Naïve Bayes (NB), Support Vector Machine (SVM), KNN, and Neural Network (NN). Table 5 shows the precision, recall, f1-score, and accuracy results of each ML model with each feature selection step. Moreover, we performed experiments considering all features (X_all). Notice that the values are filled with colors, where a green color means a higher value, and red color means a low value. XGB performs the best compared to other algorithms when observing the performance metrics such as recall, f1-score, and accuracy. Even though KNN shows 0.76 precision, the rest of the metrics show values below 0.4. Table 5 also supports that considering all the features and the chi-square method provided the best results.

**Table 5:** Testing and validation results for different models

*Model*	Feature selection	Precision	Recall	F1-score	Accuracy
*NB*	Lasso_SFM	0.68	0.52	0.59	0.52
	DT_SFM	0.63	0.64	0.63	0.64
	RF_SFM	0.63	0.64	0.63	0.64
	chi_SKB	0.67	0.63	0.64	0.63
	DT_RFE	0.67	0.65	0.65	0.65
	RF_RFE	0.66	0.65	0.65	0.65
	Lasso_RFE	0.66	0.61	0.63	0.61
	X_all	0.69	0.63	0.65	0.63
*SVM*	Lasso_SFM	0.68	0.55	0.60	0.55
	DT_SFM	0.65	0.40	0.48	0.40
	RF_SFM	0.67	0.54	0.59	0.54
	chi_SKB	0.66	0.45	0.52	0.45
	DT_RFE	0.67	0.56	0.61	0.56
	RF_RFE	0.66	0.51	0.57	0.51
	Lasso_RFE	0.67	0.56	0.61	0.56
	X_all	0.66	0.54	0.58	0.54
*KNN*	Lasso_SFM	0.76	0.29	0.38	0.29
	DT_SFM	0.65	0.54	0.58	0.54
	RF_SFM	0.65	0.53	0.58	0.53
	chi_SKB	0.65	0.54	0.58	0.54
	DT_RFE	0.67	0.57	0.61	0.57
	RF_RFE	0.66	0.55	0.59	0.55
	Lasso_RFE	0.60	0.63	0.61	0.63
	X_all	0.65	0.51	0.56	0.51
*NN*	Lasso_SFM	0.68	0.51	0.57	0.51
	DT_SFM	0.63	0.64	0.63	0.64
	RF_SFM	0.63	0.64	0.63	0.64
	chi_SKB	0.63	0.64	0.63	0.64
	DT_RFE	0.63	0.64	0.63	0.64
	RF_RFE	0.63	0.64	0.63	0.64
	Lasso_RFE	0.65	0.60	0.62	0.60
	X_all	0.63	0.64	0.63	0.64
*XGB*	Lasso_SFM	0.71	0.67	0.68	0.67
	DT_SFM	0.68	0.65	0.66	0.65
	RF_SFM	0.68	0.67	0.67	0.67
	chi_SKB	0.70	0.70	0.70	0.70
	DT_RFE	0.67	0.71	0.69	0.71
	RF_RFE	0.69	0.71	0.70	0.71
	Lasso_RFE	0.66	0.65	0.65	0.65
	X_all	0.70	0.70	0.70	0.70

In the context of this problem, it is essential to decrease the rate of mistakenly classifying an individual in higher classes when they belong to the lower class. For instance, classify a person as class 3 when they belong to class 0. Making that error could result in an embarrassing and disturbing event for the patient and their caregiver.

## Conclusion, limitations, and future work

This study developed a ML model based on XGB to predict urination times and determine important factors in the prediction model. It is modeled as a classification problem. This model could contribute to mitigating long-lasting UI problems in a data-driven manner. Using several methods for feature selection and ML, this study predicted the next urination time as a classification problem, reaching 70% accuracy. The features selected for the four classes suggest that volume and age were the most significant within the first 30 min of consuming liquids as negative impacts on urination. However, as more time persists, factors such as the number of drinks, and the volume of alcohol and water consumed become more useful as determinants in predicting the next urination. This study’s findings suggest that factors such as a child’s bladder size or an older adult’s UI can significantly impact the prediction of the next urination, which is consistent with previous research [6, 7, 24]. These results are in line with the literature finding that age can affect how much and how long a person can hold their urine [4, 6, 7, 23, 24].

This study is the first step in developing a forecast model using a feature selection algorithm and ML approaches to predict a person’s need to urinate. There were notable limitations to this research design that should be addressed in future studies. The current research design could have benefited from a larger sample, as more training data improves the overall accuracy of a ML predictive model. For example, although the number of participants is similar for each gender (28 males and 23 females), there is only one youth female participant in this study, which can affect the applicability of the outcome study to this demographic sector. Moreover, this study was performed in a specific area in Alabama, United States. Therefore, we acknowledge that the applicability of the ML model may misinterpret data from other regions from people that do not reside in the region of the study. Nevertheless, this can be fixed by collecting more data from other regions to generate a more robust model.

Additionally, replications of this study will likely lead to errors related to the accuracy of self-reported consumption and bladder voiding data. This study found that numerous participants did not accurately record their consumption and urination. These errors were more common among the child participants. Due to potential literacy or developmental gaps, children may have been less capable of accurately recording or conveying their own inputs and outputs to their parents. Future research should consider using electronic monitoring devices that can detect and record urination and consumption.

This study’s future work and testing will lead to a more precise ML model to serve as a real-time forecasting system based on an individual’s consumption pattern. This system will use the inputs of consumption and physical activities to predict the output of urination time and volume. This system could be implemented in a mobile application to make it easy for parents, patients, and caregivers to track expected urination times by entering what, when, and how much a person has been drinking. The goal of the app is to notify users when it is almost time to go to the bathroom based on previous input and output data.

## Data Availability

The datasets analyzed during the current study are available from the corresponding author on reasonable request.

## Abbreviations

UI: Urinary incontinence
XGB: Extreme gradient boosting
PFMT: Pelvic floor muscle training
SUI: Stress type UI
KNN: K-nearest neighbor
SMOTE: Synthetic minority oversampling technique
RFE: Recursive feature elimination
SFM: Select from model
DT: Decision tree
RF: Random forest
GS: Grid search
SHAP: Shapley additive explanations
